# Using magnetic particle imaging systems to localize and guide magnetic hyperthermia treatment: tracers, hardware, and future medical applications

**DOI:** 10.7150/thno.40858

**Published:** 2020-02-10

**Authors:** Prashant Chandrasekharan, Zhi Wei Tay, Daniel Hensley, Xinyi Y Zhou, Barry KL Fung, Caylin Colson, Yao Lu, Benjamin D Fellows, Quincy Huynh, Chinmoy Saayujya, Elaine Yu, Ryan Orendorff, Bo Zheng, Patrick Goodwill, Carlos Rinaldi, Steven Conolly

**Affiliations:** 1 University of California Berkeley, Department of Bioengineering, Berkeley, CA 94720, United States; 2 Magnetic Insight, Inc., Alameda, CA 94501, United States; 3 Department of Electrical Engineering and Computer Sciences, University of California, Berkeley, CA 94720, United States; 4 University of Florida, J. Crayton Pruitt Family Department of Biomedical Engineering and Department of Chemical Engineering, FL, 32611 United States

**Keywords:** Theranostics, RF hyperthermia, RF treatment planning, RF treatment monitoring, magnetic fluid hyperthermia, hyperthermia, Magnetic Particle Imaging, Magnetic Nanoparticles, Superparamagnetic Iron Oxide Nanoparticles, *in vivo* Tracking

## Abstract

Magnetic fluid hyperthermia (MFH) treatment makes use of a suspension of superparamagnetic iron oxide nanoparticles, administered systemically or locally, in combination with an externally applied alternating magnetic field, to ablate target tissue by generating heat through a process called induction. The heat generated above the mammalian euthermic temperature of 37°C induces apoptotic cell death and/or enhances the susceptibility of the target tissue to other therapies such as radiation and chemotherapy. While most hyperthermia techniques currently in development are targeted towards cancer treatment, hyperthermia is also used to treat restenosis, to remove plaques, to ablate nerves and to alleviate pain by increasing regional blood flow. While RF hyperthermia can be directed invasively towards the site of treatment, non-invasive localization of heat through induction is challenging. In this review, we discuss recent progress in the field of RF magnetic fluid hyperthermia and introduce a new diagnostic imaging modality called magnetic particle imaging that allows for a focused theranostic approach encompassing treatment planning, treatment monitoring and spatially localized inductive heating.

## Introduction

Thermal ablation is the process of increasing tissue temperature by applying heat (i.e., induced hyperthermia) to cause irreversible damage to a pathologic target. Thermal ablation is used routinely in medicine to treat heart arrhythmia, to cauterize the endometrial wall, to cauterize blood vessels, and to treat metastatic/recurrent tumors.

Numerous technologies have been developed to optimize the efficiency and localization of ablation inside the body, each with their strengths and weaknesses. One common technique, catheter-based radiofrequency (RF) ablation, utilizes an electrode mounted on a catheter to ablate nearby pathology. The catheter is placed near the pathology and utilizes RF currents in the range of 350-500 kHz to ablate local tissue. The energy is only deposited near the electrode and the size of the ablation zone is controlled by the quasistatic electromagnetic field patterns of the electrodes, with some spatial variations due to the heterogeneity of tissue conductivity and permittivity. High intensity focused ultrasound (HIFU), another common technique, utilizes ultrasound energy applied via focused ultrasound transducers to cause an increase in temperature (sometimes with direct acoustic cavitation), eventually resulting in full tissue ablation. Here the size of the treatment zone (1-3 mm) depends on the frequency of the ultrasound beam (typically below 2 MHz) and the arrangement of the transducer arrays 3, 4. Alternatively, the lesion can be cooled down to freezing or near-freezing temperature to bring about cell death as in cryotherapy.

Non-invasive imaging-guided treatment approaches are of benefit by not only localizing the tumor lesion, but also by facilitating for real-time treatment monitoring. Most common methods for imaging-guided treatments include, radiation delivery system using images from CT or PET-CT 5, 6, cryotherapy under real-time ultrasound and MRI guidance 7, 8, and HIFU under MRI guidance 9, 10.

Magnetic fluid hyperthermia (MFH), which is the use of an externally applied field to heat suspensions of iron oxide nanoparticles within the body, is a technique that has no fundamental depth limitation and has minimal heating of background tissues, owing to specific localization of the hysteretic heat to the iron nanoparticles. One of the first reported uses of MFH in medicine was for heating sentinel lymph nodes to treat metastatic tumors. Iron oxide nanoparticles were injected, such that they concentrated in the metastasized sentinel lymph nodes. Heating the nanoparticles by applying 100-300 kHz magnetic induction fields was thought to preferentially ablate metastatic tumors 11, 12. The longer wavelength of the RF fields used in MFH makes it difficult to localize the energy towards a confined location in the human body.

Iron oxide nanoparticles (SPIO) with core sizes of 4-28 nm, show superparamagnetic behavior, with magnetic saturation comparable to that of a ferromagnet but with zero coercivity and zero remanence. These nanoparticles are used clinically for iron supplement therapy in anemic patients and as a magnetic resonance imaging (MRI) contrast agent. SPIOs for MRI are used as a negative contrast imaging agent especially targeting the reticuloendothelial system 13, 14, and more recently iron oxide nanoparticles developed to treat anemia are being evaluated for off-label use as positive MR contrast agent for angiography 15, 16. MRI permits direct iron quantitation using relaxometry and susceptibility mapping approaches, however this is challenging due to the effects from magnetic field inhomogeneities, phase effect from the tissue composition and the overall ppm level sensitivity limit of the modality 17, 18.

Recently, these SPIOs have also found use in a new imaging modality, called magnetic particle imaging (MPI). Introduced by Gleich & Weizenecker in 2005 19, MPI forms images by exploiting the intrinsic saturation property of SPIOs. MPI has numerous advantages for diagnosis and therapy. MPI only sees the magnetic tracers without obscuring signal from background tissues. Also, MPI has superb sensitivity of better than 1 micromolar iron 20-23. MPI is reported to have the highest therapeutic cell tracking sensitivity of all positive-contrast imaging techniques, with a detection limit of 200 labeled cells 22. More recently, it was shown that a high sensitivity of ∼ 100 picogram per 1 mm^3^ voxel sensitivity 24 could be achieved in combination with a high temporal resolution of 46 frames-per-second, for real-time perfusion imaging of stroke lesion 25 and to evaluate blood vessel response of tumors to treatment 26. In addition to its high sensitivity and ideal contrast, MPI also has an excellent safety profile. Both the SPIO tracers and MPI scanners have no ionizing radiation, and many tracers have already been approved by the FDA or EU for safe human use 13, 27-29. The SPIO remain superparamagnetic until hydrolyzed or enzymatically degraded, as demonstrated by months-long longitudinal in *vivo* MPI cell tracking studies 30, 31. The SPIOs typically are cleared through the hepatobiliary system 14. Lastly, MPI is fully quantitative with zero depth attenuation 32, 33 and allows for the quantification without view limitations. MPI is being developed in many academic labs including from Germany 19, 34, 35, Turkey 36, Japan 37, China 38, Canada 39, 40 and the USA 31, 41-45.

**Figure 1:**
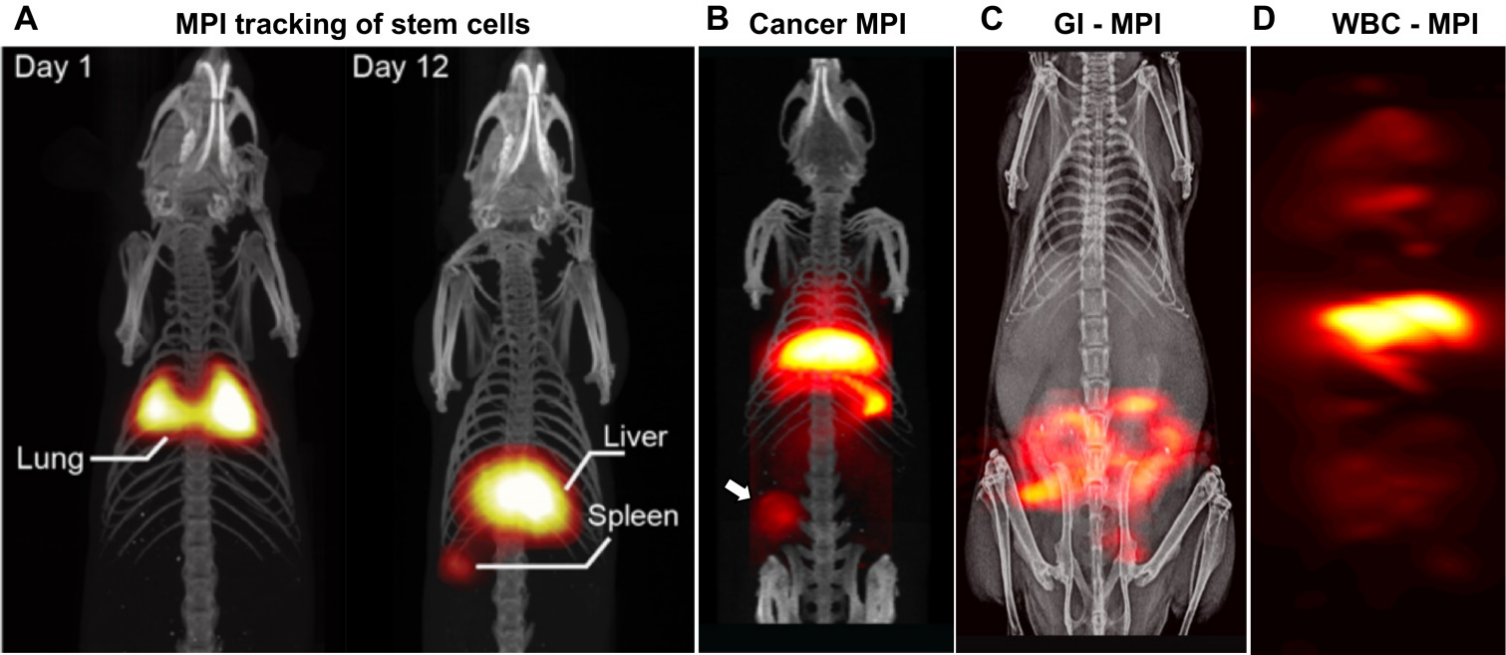
** UC Berkeley MPI Data.** (A) First in vivo tracking of stem cells using MPI, which has zero signal attenuation with depth and zero background signal (reproduced from 22 under creative common license). (B) First in vivo MPI images of triple negative breast cancer (Reproduced with permission from Yu E et al, 2 Copyright 2017 American Chemical Society) (C) First in vivo MPI study of gastrointestinal bleed assessment in a murine model, akin to Tc99m-RBC scans but without radiation (Adapted with permission from Yu E et al, 47 Copyright 2017 American Chemical Society) with 10-times greater sensitivity than RBC scintigraphy. (D) Monoclonal IgG-antibody functionalized SPIO MPI scan showing distribution of in situ labeled WBCs in the RES system of liver, spleen and marrow.

MPI complements imaging techniques of nuclear medicine. MPI tracers can be stored and used without prior preparation and lacks any complex radiochemistry. As just two examples, SPIOs targeted with macroaggregated albumin (MAA) could be injected directly from the refrigerator, with the first MPI scan in just a few minutes 46. This would be much faster than traditional Tc99m-MAA studies with Scintigraphy or SPECT, which typically require 3 hours. Similar acceleration could also be obtained using MPI for diagnosing GI bleeds 47. Several MPI applications 48, 49 have already been tested in rodent models including (see Figure 1): real-time MPI image-guidance of catheters 50, 51, stroke diagnosis 25, angiography 52, lung perfusion 46, lung ventilation 53-56, cancer imaging 2, cancer theranostics 55, 57, stem cell tracking 22, 30, 31, 44, 58, brain perfusion imaging 42, 59, magnetic hyperthermia 1, 60-62 and gut bleed detection 47.

In Magnetic Particle Imaging (MPI), an image is created by exciting the SPIOs located only at the field-free region (FFR) 63, 64. An FFR is created using strong magnetic field gradients in the order of 7Tm-1 in current preclinical scanners 2, 47. By rastering the FFR in space, SPIO distribution can be localized by the instantaneous knowledge of the FFR and the signal obtained. In a similar manner, we have recently developed a localized MFH approach, where using gradient magnetic field, heating of SPIO particles can be achieved only at the location of the FFR 1, 65, 66. The linear quantitative nature of MPI in combination with the localizing MFH treatment has great potential towards precision theranostics. In this review, we detail recent progress in RF based magnetic fluid hyperthermia (MFH) and its applications in ablation and controlled drug delivery. We will compare recent trends in image guided, *localized* MFH with MPI. Finally, we will also discuss techniques for thermometry and dosimetry for MFH.

## Introduction to MFH, SAR and PNS limits

MFH uses VLF (very low frequency) or RF (radiofrequency) magnetic fields, which induce electric fields and ionic currents within the body. Because these fields can heat or stimulate nerves in the human body, there are safe operating rules prescribed by both the EU and FDA 67, 68. In particular, there are limits to the E-field, to prevent peripheral nerve stimulation (PNS) and to Specific Absorption Rate (heating), measured in Watts per kg (W/kg).

The specific absorption rate (SAR) is a measure of the power dissipated in a biological sample, and is limited by the United States Food and Drug Administration (FDA) as described in Table 1. 69 It is defined as:


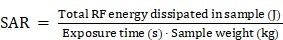
 (W/kg)

SAR is defined as the amount of energy deposited per unit mass of the tissue 70. SAR can be simplified and measured as a change in temperature (T) over a short period of RF exposure (t) (ΔT/Δt). Restricting SAR is essential to prevent a rise in the whole-body temperature. SAR can be computed using straight-forward quasi-static electric field simulators (accurate below 128 MHz) or it can be measured through calorimetric methods. SAR is often estimated *in vitro* using conductive tissue phantoms replicating the geometry of the human body. Because the specific heat of tissue is 4.2 J/(g°C), the maximum SAR limit of 4 W/kg is designed to ensure that rate of temperature rise rate remains safe.

**Figure 2:**
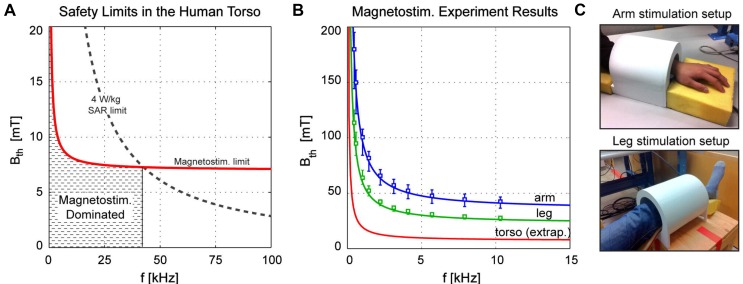
** (A) SAR & PNS** safety limit dependence on RF Frequency and amplitude as in a human torso, (B) magnetostimulation measurements in human arms and torsos 32. PNS is the dominant safety concern for MPI at drive field frequency <42 kHz. (C) Measurement setup for assessing the stimulation limits as in (B). Reproduced with permission from Saritas et al., J Magn. Reson. (2013)^32^ Copyright 2014 Elsevier.

PNS causes a sensory response in the muscle, described as a tingling, poking or twitching sensation. It is attributed to the electric field induced across neurons due to an applied oscillating magnetic field (magnetostimulation). Saritas et. al 32, 71 estimated the field amplitude and frequency dependence of the magnetostimulation effect in human arm and leg. Figure 2 explains the SAR and PNS threshold in humans. Based on the fundamental law of magnetostimulation 72, Saritas estimated that at lower frequencies of RF, the predominant safety issue in MPI is magnetostimulation. It is critical to understand the SAR and PNS limits in the design and implementation of RF hyperthermia. The readers are also referred to a comprehensive text book on RF interaction with biological material for more details 73.

## Iron Oxide Nanoparticles for Magnetic Fluid Hyperthermia

Superparamagnetic iron oxide (SPIO) nanoparticles used as tracers for MPI can also be used for MFH. The heat generated by SPIOs in response to an oscillating RF field is due to Brownian rotational or hysteretic loss of the nanoparticles and is dependent on the frequency of the oscillating magnetic field. The heating characteristics of the nanoparticles are also measured in the units of specific-absorption rate (SAR) similar to that of biological tissue heating. Intrinsic loss parameter (ILP), a property of the iron oxide nanoparticle, was pro- posed as a method to normalize SAR values measured at different magnetic field amplitudes and frequencies. The ILP is defined as the power loss dependence on the frequency and amplitude of the applied field and is defined in units of nH m^2^ kg^-1^. SAR and ILP are often interchangeably used as a measure of power deposition 74.

Rosensweig 75 provided an analytical basis for magnetic nanoparticles heating in an oscillating field. SPIO exhibit a uniaxial anisotropy, and an energy barrier exists along this axis. Magnetization of SPIO changes with the applied field with the relaxation time constant, 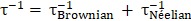. The Néel time (τ_Néelian_) constant refers to the time for the internal magnetic moment to align with the external magnetic field which is affected by the interdomain interaction within the particles, whereas the Brownian time (τ_Brownian_) constant defines the rotational time that the entire particle goes through with respect to the external magnetic field and is affected by the local micro environment. Though the Néel and Brownian relaxation coexist, in general, small core size SPIO particles can be considered as Néel relaxation dominant and large core size SPIO particles as Brownian relaxation dominant 75.

According to Rosensweig equation, the power dissipation, P, can be defined as: 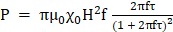 . Where, P is dependent on the relaxation time constant *τ*, applied frequency 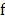, the square of the applied field amplitude H and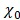, the actual chord susceptibility which in this equation corresponds to the Langevin equation. From the equation it can be observed that the heating gains can be high if the frequency is greater for *τ*.

**Figure 3:**
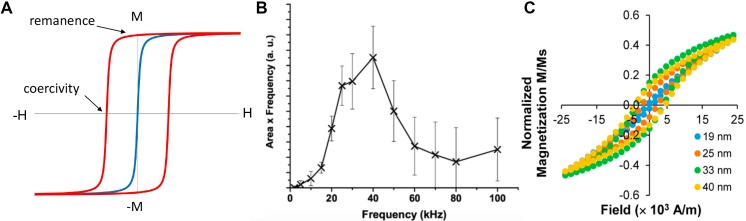
** SPIOs obey Langevin physics.** (A) Within an applied field (positive or negative) the particles align in the direction of the field. Once the field is removed the net magnetization becomes zero (blue). However, relaxation delays (similar to hysteresis) are observed when the applied field is oscillated at higher frequencies (red). Heat is generated as a result of hysteresis loss and is proportional to the area within the hysteresis loop. The heat generated in SPIO is a function of frequency and applied field. Figure (B) shows the hysteresis response of clustered Chemicell FluidmagD particles at different oscillating field frequencies. Note that the hysteric loop opens at 2 kHz. Reproduced with permission from 77 Copyright 2007 IEEE. (C) Hysteresis is dependent on the size of the SPIO particles and greater heating can be achieved with larger particles. Adapted with permission from Tong S et al., ACS Nano. 81. Copyright 2017 American Chemical Society.

At higher frequencies of applied external fields, the SPIO particles show hysteresis. The frequency below which a particle behaves superparamagnetic is referred to as the blocking frequency 76, 77. Above the blocking frequency, heating due to hysteresis loss is observed, as shown in Figure 3. The blocking frequency and heating characteristics are influenced by the particle size, shape, particle dispersity and the viscosity of the surrounding media.

Tay et al. used 13-nm core iron oxide nanoparticles coated with poly-ethylene glycol and reported no rise in temperature of the solution containing particles subjected to a 20 mT, 20 kHz alternating magnetic field (a frequency typically used for imaging in MPI). The same particles demonstrated a SAR of 120 W/kg of tissue at a 354 kHz and 13 mT field amplitude 1.

Shape anisotropy in nanoparticles can improve their heating properties 78, 79. Khurshid et al. reported a 1.4-fold improvement in the particle SAR for cubic iron oxide nanoparticles compared to spherical particles 80. The cubic particles had a 1.5-fold increase in effective anisotropy field compared to spherical particles. SAR improvement of nanoparticles can also be achieved by controlling their size. Sheng Tong et al. demonstrated the hysteresis loss of nanoparticles increases with particle size from 11-33 nm and saturates at sizes > 33 nm 81. Aggregation of nanoparticles can also result in better heating performance due to increased anisotropy resulting from dipole interaction from between the neighboring particles under the influence of the applied field 82-86. Doping ferrite with anisotropic material such as cobalt, zinc, nickel or manganese can also improve the heating property of the materials by increasing the overall anisotropy 87-89.

Rosensweig 75 further discussed the effect of viscosity and particle dispersity on the heating characteristics of SPIO. The Brownian relaxation constant (in the case of large size particle) especially seems to be strongly dependent on the viscosity of the matrix fluid, with better heating characteristics with increasing excitation field frequency at a given viscosity. Whereas, polydispersity can degrade heat production of the nanoparticle. Dennis CL & Ivkov R, in their work 90 derive the effect of polydispersity from the Chantrell equation 91, where by accounting for polydispersity in the lognormal distribution of particles, the heat generated can be assumed to be distributed broadly over the size distribution of the particles. There appears to be a scarcity on experimental work reporting the influence of viscosity and polydispersity of SPIO for MFH 90, 92. This can be attributed to the complex interplay of various nanoparticle parameters such as zeta potential, surface coating, solvation chemistry, configuration of the particle coating and particle-protein interaction; all of which can have a profound effect on the colloidal stability of the particle system.

Coating material apart from imparting biocompatibility on SPIO can also affect the heating performance. To a first degree Jordan A et al., 93 noticed a difference in SAR between dextran coated and aminosilan coated iron oxide nanoparticles, with aminosilan particles having 1.2 fold better SAR than dextran coated particles, with silane coated showing better malignant tumor cell labeling. Liu XL et al., in their work 94, performed a systemic analysis of SAR using SPIO coated with different molecular weight PEG, and observed an increase in SAR with decrease in the thickness of the surface coating, attributing to the dominance of Brownian relaxation based heat losses. However, the reduced thickness can also affect the colloidal stability of the particles that is also affecting the SAR measurement. Further, the heating performance was also noticed to depend on the thermal conductivity of the coating 85, 94. The effect of coating material and SAR was also observed to have intrinsic dependence in the solvent in which the particles were suspended, the zeta potential defining the colloidal stability, and the amount of the coating material around the nanoparticles.

MPI resolution and sensitivity is also dependent on the SPIO characteristics, with 24-28 nm core size SPIO particle providing the optimal resolution and sensitivity for MPI, any further increase in size caused blurring of images in MPI 95. Currently, the LodeSpin particles provide the best resolution in the order of 0.8 mm and detection sensitivity of about 3 ng of Fe 48. The effect of MPI drive field amplitude and frequency on the particle size ranging from 18-32 nm was investigated by Tay ZW et al 96. In this work, a systematic optimization effort was carried out to identify ideal operational conditions for MPI resolution and sensitivity characteristics over a range of nanoparticle sizes. A striking difference was observed between two groups of particle (a) small core size particles (18-24 nm) and (b) large core size particles (27 & 32 nm) with two optimal operation conditions, 70 kHz and 5 mT (for smaller core particles) and 1 kHz and 14 mT (for large core particles).

MPI images are acquired in partial field-of-view segments 33, 71, 97, as a result of which the particles do not heat up, unlike continuous excitation in MFH. Also, new pulsed MPI approaches can mitigate hysteresis loss from SPIO particles 98. We propose a size range of 24-28 nm would be optimal for both MPI and MFH. Further SPIO configuration such as chaining and aggregation that increases the anisotropy would be interesting to evaluate for combined MPI and MFH. On the other hand, smaller size particle might provide poorer resolution and sensitivity in MPI and weaker hysteresis loss for MFH 99.

**Figure 4:**
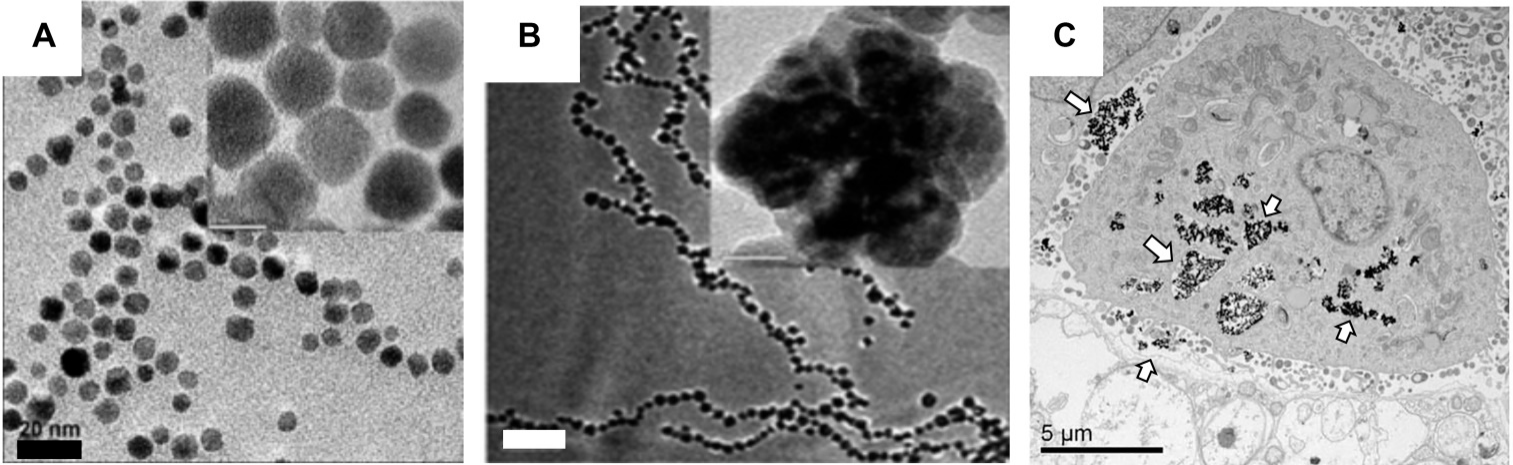
** Clinically used SPIO for MFH consist** of aminosilane surface modified 10 nm iron oxide nanoparticles. (A) TEM images of 3-aminopropyltriethoxysilane coated iron oxide particles that are highly mono-disperse (scale bar = 20 nm)(Reproduced with permission from 101 Copyright 2010 IEEE) (B) Aggregated and chained nanoparticle configurations can improve the effective SAR value (500 W g^-1^ at 89 kA m^-1^ AC magnetic field and 240 kHz frequency)) (scale bar = 200 nm). Reproduced with permission from 102 Copyright 2011 The Royal Society of Chemistry. (C) TEM images reveal the aminosilane coated nanoparticles are endocytosed and adhere strongly on the surface of the cancerous cells (arrows), greatly increasing the chances of localizing the heat (scale bar = 5 µm) 103, 104 (Reproduced with permission from 106 Copyright 2019 Elsevier).

Delivering adequate dosage of nanoparticles to tumors for MFH is still particularly challenging, with almost all i.v. administered nanoparticle system relying on the enhanced permeation and retention phenomenon (EPR) of the tumor for targeting, of which only 0.7% median dose of nanoparticles makes its way into the tumor vicinity 100. Since SAR is a function of the concentration of SPIO particles in MFH, large quantities of SPIO must be present in the vicinity of the tumor. This is achieved by an intra-tumoral administration of the prepared nanoparticles. Currently, NanoTherm® iron oxide nanoparticles produced by MagForce Nanotechnologies GmBH are available for clinical use in the European Union. NanoTherm® is composed of 12 nm core size nanoparticles with a bio-compatible aminosilane coating. The solution is available at a very high concentration of 112 mg/mL of iron 101 relative to Feridex *i.v.* solution's concentration of 11.2 mg Fe/mL 102. The difference in concentrations of the parenteral preparation for the therapeutic versus diagnostic purposes is because a large localized concentration of iron oxide is required to achieve reasonable heating to ablate tissues. For MFH, the particles are administered locally by a stereotaxic procedure and the aminosilane coating ensures strong interaction between the target tissue and particles, Figure 4C.

## MFH treatment

Given the safety limits of RF fields imposed by human SAR and PNS, it is common for MFH researchers to employ relatively high concentrations of nanoparticles to allow for heating of the target tissue. To achieve a therapeutic response in tissues using hyperthermia, two treatment approaches are used. In the first approach the temperature of the target tissue is raised to 40-45°C and held for a defined amount of time causing changes and cellular death, and in the second approach the tissue is completely ablated at a higher temperature of 75-90°C.

Ablation techniques are used in the clinics and mostly applied locally using a catheter-based approach. Ablation technique requires short tissue exposure time to high temperatures and can lead to what is referred to as coagulative necrosis. Apart from RF based ablation approach, focused ultrasound achieves localized temperature of ablation. A whole-body exposure to temperatures of ablation will not be tolerable by humans.

A biological material can be considered as a dielectric media comprising of large amount of water, ions (sodium, potassium and calcium) and macromolecules (proteins, fat and com- plex sugar). Heat from hyperthermia can cause molecular and physiological change in the tissue. At the molecular level, heating causes conformational changes in macromolecular proteins and DNA resulting in impaired cellular function, causing senescence, apoptosis and necrosis 105, 106. Heat shock proteins, that are over expressed in cells during physical stress and injury, including exposure to hyperthermia, elicit anti-tumor activity 107. There is evidence that an increase in infiltration of antigen presenting cells occurs during hyperthermia, causing cytotoxic immune responses in the tumor 108. Moreover, other physiological changes in tumor such as an increase in blood perfusion and altered metabolism, as a result of hyperthermia can sensitize tumor for combinatorial therapy such as chemotherapy and radiation therapy 109. Newer research suggest that the therapeutic response due to RF field may be brought about by mechanically actuated deformation and shredding of SPIO bound proteins in the intra-cellular milieu of cancer cells 110, 111. This would require a sub-optimal rise in temperature and greatly reduce the exposure of patient to RF excitation. The underlying mechanism of biological cell death as a result of shredding requires further investigation.

To measure the effective thermal dose delivered via hyperthermia, the cumulative equivalent minutes at 43°C (CEM43°C) was proposed in 1984 by Sapareto and Dewey 112, 113. The CEM43°C allowed for the standardization of thermal dose across publications to a common unit by converting any time-temperature history into equivalent number of minutes of heating at 43°C and is defined as, 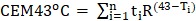, where *t_i_* is the i-th time interval, T is the average temperature during time interval ti and R is related to the temperature dependence of the rate of cell death (R(T < 43°C)=1/4, R(T> 43°C)=1/2). R is a measure of several factors associated with cell death and is influenced by the parameters discussed above.

The effect of heat in biological tissue is comparable to that of radiation exposure 114. The surrounding pH of the target tissue, the metabolic state and blood flow (hypoxic versus normoxic) conditions all affect the response of the tissue to heat. In certain circumstances, the tissue can generate a thermotolerance: a state where the cells become resistant to heat. Heat shock protein provides protection to the cells and tissue during physical stress. Repeated exposure to physical stress will allow for tolerance development. The heat shock proteins act specifically by managing protein synthesis, allowing for permeability changes to resist heat exposure, and reduce cytokine related stress mediated inflammation 115-117. Mechanistically the thermotolerance was related to the expression, accumulation and relative half-lives of the heat shock proteins. The tissue of interest must lose the tolerance before it can be treated.

Thermotolerance is a key issue during MFH, which reduces effective killing by 4-10 folds. Cells take up to 100 hours to lose their sensitivity to thermotolerance 112-114. It is estimated that to effectively treat a tumor, at least 90% of the tumor should receive a CEM43 of 25 118. Thus, a long duration of hyperthermia is needed in order to achieve effective treatment. To reduce the duration of treatment, MFH is administered in combination with radiation therapy. Heating improves the sensitivity of the cancer cells to radiation and reduces the treatment time needed 119.

## Clinical Systems for MFH

Clinical MFH based pilot studies have been carried out in treating prostate and recurrent brain tumors in humans. The first reported use of MFH, was in a patient unresponsive to high dose brachytherapy for a T3 prostate carcinoma 120. The overall treatment procedure was divided into three parts. The treatment of solid prostate tumor was first evaluated using non-invasive CT imaging. Treatment planning was then carried out based on the CT images. Parameters such as the particle dose needed, the site of particle placement, and the corresponding SAR with the applied field were estimated using simple numerical methods, that accounted for tumor margins, environment surrounding the tumor and blood supply to the tumor (that can act as a heat sink). Finally, a dose of 12.5 mL of iron oxide fluid was injected into 35 mL prostate organ using a trans-urethral catheter under ultrasound guidance. Hyperthermia was applied using an RF applicator. The authors of this clinical study noticed patient discomfort and pain during RF application and had to temporarily reduce the applied field.

In another clinical evaluation, MFH was carried out in combination with radiation therapy in treating an aggressive recurrent brain tumor (glioblastoma) 119, once again divided into three parts. In the first part, the patients underwent surgery to remove tumors and had SPIO particles administered stereotactically. Secondly, MRI and CT images were acquired to image the particle distribution and a forward model was applied to estimate the heating boundaries. Finally, combined MFH and radiation therapy was used for treatment.

In both clinical studies, hyperthermia treatment was carried out with an implanted thermometric probe to measure the heat generated 121. The latter clinical trial reported no side effects and in combination with radiation improved the overall survival of patients with first recurrence of the brain tumors.

The clinical magnetic hyperthermia system used in the glioblastoma clinical study was built at MFH Hyperthermiesysteme GmbH. While technical information about the clinical hyperthermia systems used is limited, there are several papers describing how the clinical systems were developed 122, 123. The largest difference between a small animal system and clinical system is the coils to generate the alternating magnetic fields required for MFH. In a small animal system, both air-core and ferromagnetic-core coils can be used to generate alternating magnetic fields 123. However, air-core coils cannot be used in clinical scanners due to the size of the operating area, as generating sufficient magnetic field strength over large areas using these cores requires excessive power.

**Figure 5:**
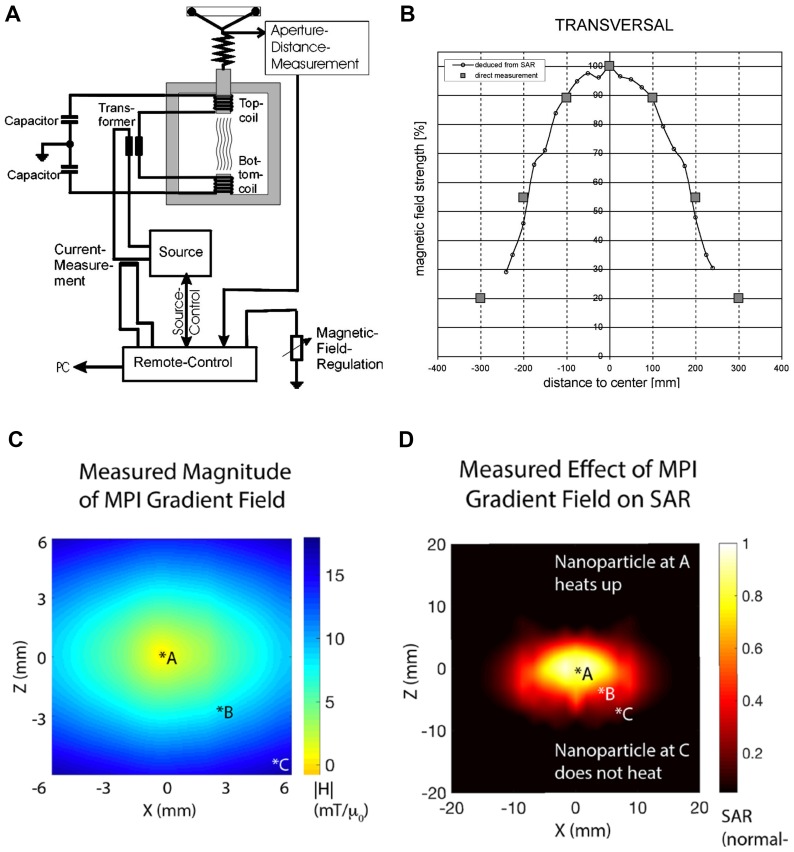
** Commercial hyperthermia systems:** (A) A clinical MFH 300F hyperthermia system 122 consists of a resonating circuit at a ferrite yoke that generate an oscillating field of 100 kHz between the pole shoes above and beneath the treatment aperture. The setup can be adjusted to limit the exposure of oscillating magnetic field exposure on the patient and (B) its corresponding field homogeneity in the transverse direction 122. The patient is slid through the aperture and the aperture distance controls the effective area of exposure and the field can be adjusted by adjusting the current to the coils. (Reproduced with Permission from 122, Copyright 2004 Wiley & sons) (C) Localized hyperthermia system with 2.3T/m field-free line gradient developed using permanent magnets at UC Berkeley 1, 60, and (D) its measured heating area of 7 mm diameter. The particles in zone *A heat up the most, with steep fall off at zones *B and *C. (Reproduced with permission from 1, Copyright 2018 American Chemical Society).

The high current and voltage in air-core coils also introduce safety concerns 123. Hence, in clinical systems ferromagnetic-core coils are used to minimize the power consumption and lower the power and voltage across the coils. The schematic of a clinical magnetic hyperthermia system is shown in Figure 5.

The magnetic field in the operation area is generated by two ferromagnetic-core coils (Figure 5 A-B). The field strength is adjustable from 0 to 15 kA/m corresponding to 0 to 20 mT 122. Homogeneity of the magnetic field is essential for equal heating of the treatment area. The clinical systems can realize 90% transverse homogeneity within a 20 cm range as shown in Figure 5B 123.

As discussed previously, clinical studies have strongly relied on CT images and MRI for estimating the nanoparticle distribution and for treatment planning. These imaging modalities have difficulty in accurately estimating treatment SAR. The difficulty with using X-ray CT for visualizing iron oxide is that, a considerably high iron content will be needed for any discernible signal in CT, whereas higher iron oxide nanoparticles concentration in MRI cannot be accurately estimated 104, 120, 122.

## Introduction to Magnetic Particle Imaging for localized MFH

**Figure 6:**
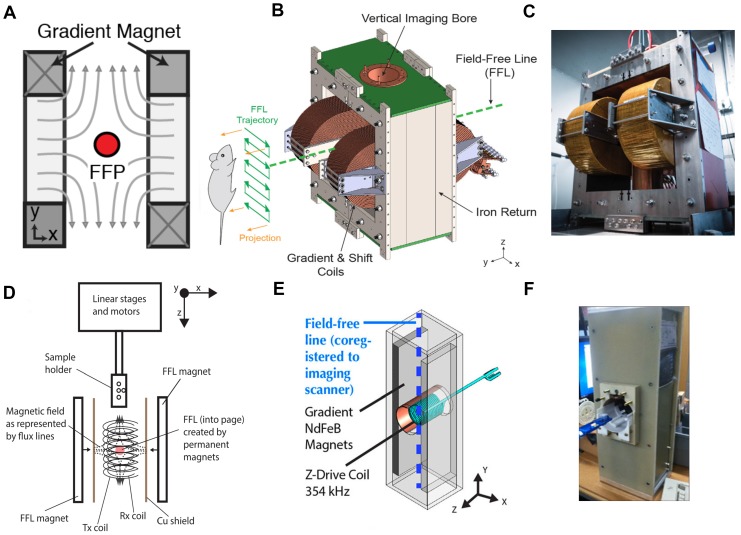
** Magnetic Particle Imaging (MPI)** uses a (A) gradient magnet to create a field-free-region (FFR). The FFR can be a point (field-free point or FFP) or a line (field-free line or FFL) in space. Magnetic nanoparticles at the FFR are free to rotate and oscillate in response to low (20-40 kHz, very low frequency range) excitation fields, while particles away from the FFR are locked due to the magnetic field. The induced magnetization from the particles in the FFR can be picked up by sensitive coils using Faraday's principle of induction. By rastering the FFR across the entire field-of-view, an image indicating the quantity and spatial location of the nanoparticle tracer can be produced. (B) An engineering design diagram of an FFL MPI scanner and an MPI scanner at UC Berkeley 47 (Reproduced with permission from Yu E et al, Copyright 2017 American Chemical Society)(C) the UC Berkeley FFL scanner has two electromagnets, and the FFL trajectory can be rastered in space by controlling the current through the electromagnets. 3D images can be obtained using tomographic reconstruction algorithms. (Reproduced with permission from Yu E et al, Copyright 2017 American Chemical Society) (D) The same principle of MPI can be applied to localize particle heating (spatially controlled Magnetic Fluid Hyperthermia) only at the FFR. Using a gradient magnet and RF coil operating at higher excitation frequency (354 kHz), spatial control of particle excitation can be achieved (Reproduced with permission from 60, IOP Publishing. Copyright Institute of Physics and Engineering in Medicine. All rights reserved) (E) & (F) engineering design diagram and an image of a field-free-line Magnetic Hyperthermia setup at UC Berkeley. (Reproduced with permission from 1, Copyright 2018 American Chemical Society).

MPI is a new and evolving tracer imaging modality with distinct imaging physics from all other imaging modalities—including MRI. MPI images the intense electronic magnetization of SPIOs (600 mT), which is 100-million-fold more intense than the nuclear paramagnetism of water at 1.5T, which has nuclear susceptibility 3.8 ppb 124. Importantly, it is not possible to obtain an MPI scan within an MRI scanner. Instead, an MPI scanner uses a very strong gradient magnetic field (current preclinical scanners use up to 7 T/m) to generate a field-free region (FFR) as shown in Figure 6 that is rastered across in space to generate images of superparamagnetic iron oxide nanoparticle tracers. The FFR occurs at the center of two or more magnets designed to create a field gradient.

Within the FFR, SPIO particles experience minimal magnetic fields, and are free to rotate under the influence of a weaker 20-40 kHz excitation field. Outside of the FFR, particles are locked to the strong fields lines of the gradient and are hence unable to rotate. In combination with the FFR and the Langevin behavior of the SPIO, it is possible to create an image of the location of SPIO in space 2, 19, 32, 33, 47, 64, 125.

The homogeneity requirements of MPI are modest: about 1% in-homogeneity is well tolerated in the selection field gradient, which is several orders of magnitude less stringent than the 1 ppm homogeneity required for MRI. Indeed, this explains why MPI images of the lung are completely unaffected by the 10 ppm field variations due to air-tissue interfaces. Many MRI pulse sequences fail in the lung due to short T2* times.

A long-standing challenge in the MFH field is localizing the heating to a precise target deep within the body. This is important because the nonspecific uptake of SPIOs to the liver, kidney and spleen typically exceeds the specific uptake to the tumor. Unfortunately, it is not possible to focus 300 kHz magnetic fields to avoid heating the liver, because the wavelength of 300 kHz magnetic fields is longer than 100 meters in vivo. Hence, current techniques for MFH pose a risk of heating the liver when we wish to ablate only a tumor. To address this important technical challenge, we exploited the MPI gradient field to localize MFH heating 1, 60, 65, 66. Here, despite using a 300 kHz magnetic field, we were able to localize heating to a small region of space, roughly the size of a grain of rice or just a few millimeters in size. Akin to MPI, we used a gradient magnetic field to prevent heating of SPIOs outside the FFR region. Only the SPIOs inside the FFR can rotate and thereby undergo induction heating, as shown in Figure 6.

## Recent Innovations using combined MPI-MFH

Magnetic particle imaging has opened a new venue for localized MFH treatment. The field-free region (FFR) used to generate images in MPI can also be used to spatially localize heat only at the point of FFR with minimal heating elsewhere. Dhavalikar et al. discussed the theoretical basis of localized MFH using FFR 66. Bauer et al., first demonstrated localized heating of nanoparticles in 1D using a gradient field generated with a permanent magnet 41. While, Hensley & Tay et al. 1, 60, 65 developed the first 2.3 T/m magnetic gradient localized MFH system, capable of selectively heating SPIO particles separated by a distance of 7 mm (Figure 6E & F). The localized MFH system consists of two permanent magnets with poles facing each other resulting in a field free region (Figure 5C). The field free region can be moved anywhere within the patient and selective heating can be achieved. The size of the FFR can be varied by varying the strength of the magnetic field gradient. Thereby catering to the size of the lesion to be treated. A water-cooled copper solenoid coil was used to excite the nanoparticle at frequencies of 300kHz to generate heat. The measured SAR in the field gradient for a 11 nm SPIO particles coated with polyethylene glycol is shown in Figure 5D. From Figure 5D it can be observed that the heat from the particle is confined to a zone of 7 mm in diameter.

MFH requires robust treatment planning and it is essential to quantify the particle deposits at the site of the tumor for accurate SAR prediction and for subsequent treatment 123. MPI has a broad dynamic range and is able to detect and quantify SPIO particles from few nanograms to several milligrams 30, 97.

**Figure 7:**
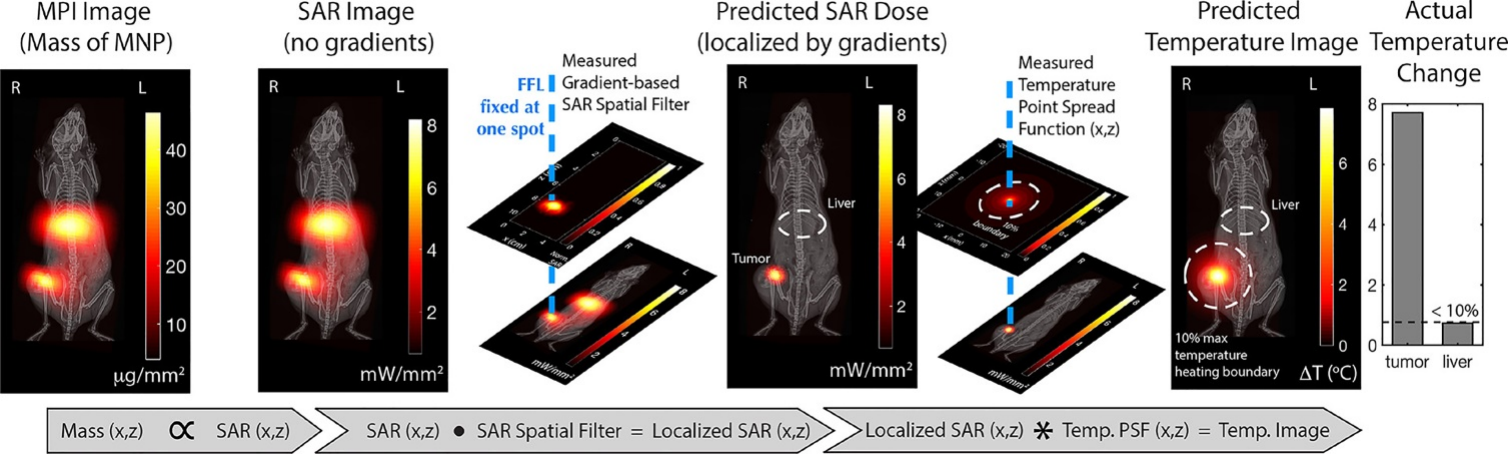
** MPI image guided MFH Treatment design planning** proposed by Tay et al. 1 MPI enables precise spatial quantification of the amount of SPIO particles administered and the gradient system enables control of the spatial zone to which RF is applied. Based on a prior knowledge of the particle response to the field, a temperature map can be generated. (Reproduced with permission from 1, Copyright 2018 American Chemical Society).

**Figure 8:**
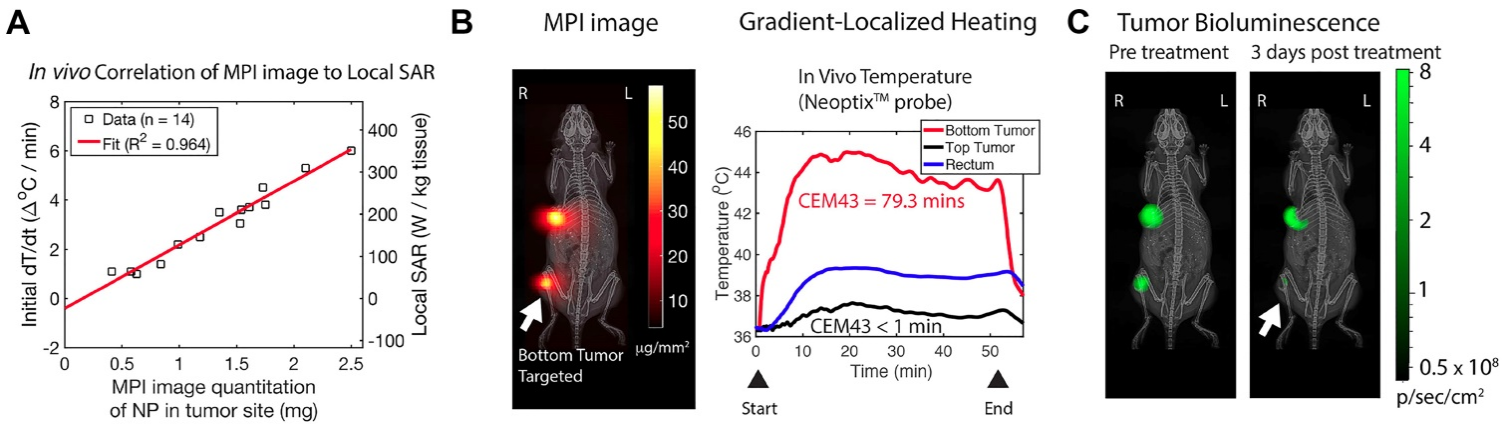
** In *vivo* localized MPI guided MFH experiment.** (A) MPI images provided quantitative information of SAR estimate at the tumor site Tay ZW et al. 1 (B) Using a 2.3 T/m magnetic field gradients, the FFR was localized towards the bottom tumor of a dual tumor xenograft mouse model and (C) a thermal probe was used to estimate CEM43 of 80 minutes that was achieved using RF excitation of the nanoparticles. (D) The bio-luminescence activity after treatment confirmed selective growth inhibition of the treated bottom tumor compared to untreated top tumor. (Reproduced with permission from 1, Copyright 2018 American Chemical Society).

In our recent work, MFH guided through MPI was studied *in vivo* 1. MPI can accurately quantify the amount of iron oxide administered and predict the amount of thermal dose that can be deposited in the target tissue using a forward model workflow (Figure 7) 1. The SAR estimate was linear with the mass of the iron oxide nanoparticles, Figure 8A. Once the particle distribution and SAR are predicted, a spatial filter can then be applied, by moving the FFR in space to the appropriate location, at which hyperthermia is to be applied. Finally, the RF excitation can be tuned and controlled based on the temperature change required and maintained for adequate heating.

The workflow described above was evaluated in a murine model with dual tumor xenograft of human origin. Figure 8B shows the in vivo distribution of iron oxide nanoparticles that were intratumorally injected and imaged using MPI. The FFR was positioned on one of the tumors (bottom) and was heated to a CEM43 of 79.3 minutes (measured using an optical fiber thermal probe) while the particles in the other tumor (top) did not respond to RF excitation. The overall body temperature of the mouse was not affected (based on rectal temperature measurement). The treatment was confirmed by a reduced bio-luminescent activity indicative of tissue death, Figure 8C. This demonstrated the localized treatment of tumors, while sparing other tissues like the liver, which typically shows high non-specific SPIO uptake 1, 60. Though, it is unclear at this stage if the RF interaction with biological tissue outside the FFR can cause burns, this first study did not report any side effects nor any non-specific rise in temperature in the murine model as a result of applied RF fields.

Recently, newer designs for the FFR are being investigated 126 that can be tuned to cater the shape of the tumor to be treated. MPI guided MFH was reported in another study by Du et al. The authors achieved tumor targeting and particle retention by targeting the fibrin-fibronectin complexes, that are over expressed in tumor interstitium, using SPIO with surface functionalized small molecule peptide, CREKA 38. They also reported a quantitative comparison between MRI and MPI. MRI was found to have much more difficulty in quantifying iron oxide tracers than MPI, as iron oxide tracers generated negative contrast in MRI versus positive signal in MPI. Finally, the authors were able to ablate the tumor using RF excitation.

Treatment dose in MFH is monitored as a function of rise in temperature. Current techniques involve the use of optical-fiber probes or infra-red cameras to measure the temperature changes. The latter is used when positioning the optical fiber probe is precluded by difficulty and invasiveness. Non-invasive imaging tools are being evaluated for treatment monitoring during hyperthermia. A rich area of innovation is measuring the temperature change during hyperthermia treatment, as it is of utmost importance for safe and effective treatment.

MRI can measure temperature changes based on a subtle shift in proton resonance frequency (10 ppb/degree C) 127-129. MRI thermometry has been combined with HIFU for cancer theranostics and for pain management 128, 130. With high-resolution spatio-temporal information from MRI and the ability for real time monitoring, MRI can help to precisely determine HIFU treatment and monitor its course.

It is possible to extend MPI technique towards real-time temperature monitoring. SPIOs used in MPI and MFH, respond to an RF excitation through a relaxation process dependent on the dominant behavior of the particles, i.e., Brownian and N´eel relaxation. Brownian relaxation can be considered as the time for the entire particle to rotate or flip in response to the excitation field and is influenced by the immediate surrounding or the micro-environment of the particles, while N´eel relaxation can be considered as a time constant for the internal flip in magnetization of the particles under the influence of the excitation field without any physical rotation 131-133. The relaxation times of SPIOs may provide information about the temperature or viscosity of the surrounding fluid in the vicinity of the SPIO 36, 51, 134, 135. Recently Weaver et al. proposed a method to measure the local micro-environment temperature of the administered SPIO, by correlating the relaxation of the SPIO as a monotonic function of temperature 136. In another work, Pantke et al. proposed a multi-frequency excitation approach to measure temperature and viscosity of SPIO environment 137. Combined MPI-MFH systems may soon permit thermal monitoring and dosimetry before, during and after MFH treatment. While measured viscosity changes can be correlated to the degree of ablated tissue death that is brought about after treatment 138.

**Figure 9:**
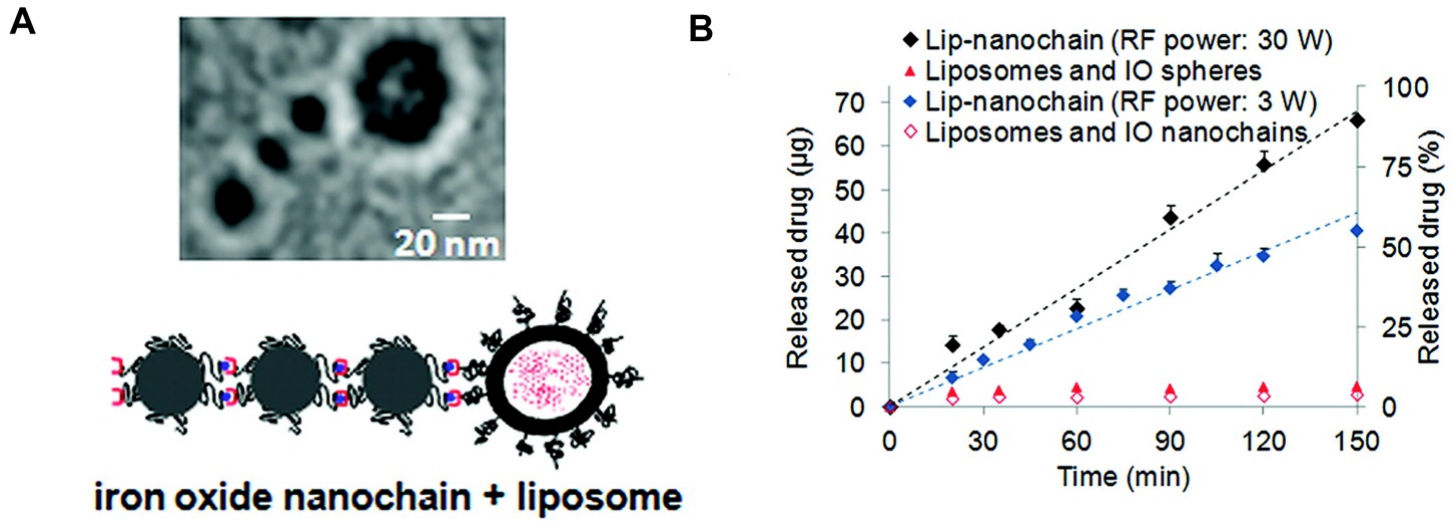
** Newer theranostic approaches exploiting SPIO** response to RF for controlled drug release. Oscillation from RF fields can be used to actuate drug release from a chained nanoparticle system. (Adapted with permission from 141, ACS Nano (2012). Copyright 2012 American Chemical Society.)

More recently, newer approaches in combining MFH to modulate drug delivery were explored 92. This is achieved by a multi-functional nanoparticle design, comprised of SPIOs and a drug of interest, within a polymer matrix with thermo-labile chemical cross-links 139, 140. The thermally labile bond breaks due to localized heated generated by SPIOs in response to RF excitation, eluting drug in the process. The process of RF actuated drug delivery requires reduced exposure of RF to the subject, thereby greatly reducing the SAR effect, while the controlled drug release improves drug efficacy. Peiris et al, developed a SPIO based nanoparticle chain assembly (shown in Figure 9) that mechanically oscillates when subjected to RF field, shredding the nanocarrier and allowing for controlled release of drug from the chain-assembly 141.

Similar to using an FFR for spatially localizing heat, few researchers have also attempted to spatially control the release of drug using FFR generated with a gradient magnetic field 142, 143. Fuller et al. developed a thermo-labile drug co-polymer conjugate encapsulating iron oxide nanoparticle, and provided a proof-of-concept spatially controlled drug release using an FFR generated using a 1.27 T/m magnetic field gradient 143. In another study, iron oxide nanoparticles were co-encapsulated in liposomes (containing the drug of choice), of a low phase transition temperature. Magnetic hyperthermia from RF oscillation resulted in an increase in temperature causing a phase transition in the liposome and selectively triggering drug elution, within a target region of ∼3.2 mm radius 142. Spatially localized heat generation and release of drug, apart from providing combinatorial effect in treating tumors, will also be invaluable for reducing the systemic toxicity of the drug.

Another theranostic approach is to selectively maneuver SPIOs using gradient magnetic field towards the target tissue. For instance, SPIOs delivered to lungs as inhaled-aerosol can be additionally moved in an image-guided fashion to a target region of the lung (for example a tumor lesion), thus improving therapeutic efficacy 53, 55, 61, 144.

Apart from localized heat deposition and drug delivery, there is a possibility for gradient based localized MFH approach to be extended for neuromodulation through remote excitation of implanted SPIO. The heat generated from the RF excitation of SPIO can trigger genetically modified heat sensitive protein transporter channels in the neurons, that can elicit an action potential 145-147.

MPI in combination with MFH and carefully engineered nanoparticles can provide superb sensitivity and contrast as well as precise actuation of magnetic-based therapies and will be a promising theranostic platform.

## Discussion & Conclusion

Image guided treatment techniques have shown effectiveness in improving precision in localizing the treatment. Image guided RF ablation technique and focused ultrasound are currently in development for treating lesions. While RF hyperthermia technique is limited due to the difficulty to focus (*λ*/2 ∼ 50 m) 73, focused ultrasound can be difficult to be implemented in region such as brain due to sound reflective property of the skull 148.

Magnetic Fluid Hyperthermia (MFH) on the other hand has been in development for many years but has just recently come into increasing clinical use. Moreover, novel innovations are rapidly increasing the efficacy of the technique. MFH is currently FDA approved in the US, with the completion of stage 1 enrollment of 120 patients. A follow up single-arm study has been proposed for focal ablation of solid prostate cancer that will be conducted at University of Texas, San Antonio and University of Seattle, Washington 149.

MPI is a novel and developing imaging modality that has already found complementary usefulness in diagnosing various disease ailments. Currently, only preclinical MPI scanners are available through Bruker GmbH and Magnetic Insight Inc, enabling its use in a vast array of biomedical research. Innovations such a Magnetic Particle Imaging (MPI) can streamline the treatment protocol by complementing MFH through treatment planning including dosimetry, thermometry and post-treatment monitoring of the tumor. MPI now allows for well-localized MFH, removing the concern for non-specific deposition of heat. However, realization of a clinical scanner requires addressing both the regulatory and safety requirements. Scaling up an MPI-MFH system will require higher power and increased cost. MPI-MFH tailored SPIOs with high resolution and sensitivity with efficient heating performance would require rigorous screening for FDA approval 28. Tissue SAR due to RF excitation needs careful consideration while performing MFH. A cooling blanket around a patient can mitigate heating during MFH procedure. Further, delivering adequate amount of SPIO to tumor non-invasively across various physiological barriers for MFH is challenging, with almost all MFH procedure being carried out with intra-tumoral delivery of nanoparticles. Newer magnetic field gradient based approaches under investigation, can provide improved delivery of SPIO particles to tumor by creating a radial force that can carry the particle across the tumor endothelium 150, 151.

With added benefit of MPI for MFH, FDA approval for MPI can be fast tracked for theranostics through specialized consideration depending on the overall survival benefit of the MPI guided MFH treatment. Clinically translatable prototypes of MPI scanners are currently being designed and reviewed 152, 153. Further, approaches with tunable field-free region can provide better control and precision in heat deposition. Novel nanoparticle design, can facilitate efficient targeting of tumor required for MFH, and other newer combinatorial approaches can give rise to safer alternatives for treatment of cancer 154-157.

## Figures and Tables

**Table 1:** FDA SAR limits for each organ. 69

Tissue	FDA SAR Limit
whole body	0.4 W/kg
head	3.2 W/kg
any 1 g of tissue	8.0 W/kg

## Abbreviations

MPI: Magnetic Particle Imaging
SPIO: Superparamagnetic iron-oxide nanoparticles
CT: Computed Tomography
MRI: Magnetic Resonance Imaging
FFR: field-free-region
HIFU: high-intensity focused ultrasound
US: Ultrasound
FDA: Food and Drug Administration
EU: European Union
FFL: field-free line
FOV: field-of-view
PSF: point-spread-function
SAR: Specific absorption rate
PNS: Peripheral Nerve Stimulation
ILP: Intrinsic loss of parameter
CEM43: cumulative equivalent minutes
RES: reticuloendothelial system
WBC: white blood cells
